# On the Morphology of the *Drosophila* Heart

**DOI:** 10.3390/jcdd3020015

**Published:** 2016-04-12

**Authors:** Barbara Rotstein, Achim Paululat

**Affiliations:** Department of Zoology/Developmental Biology, University of Osnabrück, Osnabrück 49069, Germany; barbara.rotstein@biologie.uni-osnabrueck.de

**Keywords:** *Drosophila melanogaster*, cardiovascular system, heart, dorsal vessel, circulatory system

## Abstract

The circulatory system of *Drosophila* *melanogaster* represents an easily amenable genetic model whose analysis at different levels, *i.e.*, from single molecules up to functional anatomy, has provided new insights into general aspects of cardiogenesis, heart physiology and cardiac aging, to name a few examples. In recent years, the *Drosophila* heart has also attracted the attention of researchers in the field of biomedicine. This development is mainly due to the fact that several genes causing human heart disease are also present in *Drosophila*, where they play the same or similar roles in heart development, maintenance or physiology as their respective counterparts in humans. This review will attempt to briefly introduce the anatomy of the *Drosophila* circulatory system and then focus on the different cell types and non-cellular tissue that constitute the heart.

## 1. The Circulatory System of Flies

Studying organogenesis and organ functionality requires basic knowledge of the functional anatomy of the organ itself. Herein, we will provide an introduction to *Drosophila* heart anatomy that will guide the reader through the very basic principles of *Drosophila* heart function and the cell types that constitute the cardiovascular system of the fly. Our review is especially aimed toward researchers who do not work primarily with the *Drosophila* model system. Therefore, we will not discuss the histology and morphogenesis of the heart in detail; instead, we will present a general overview, with selected histological data illustrating the *Drosophila* heart architecture. Moreover, the pictures shown were chosen as representatives of the available imaging methods that are widely used in the *Drosophila* system and that have been developed over the years since the seminal anatomical studies of Miller and Rizki [1,2].

## 2. Low and High Hydrostatic Pressure Circulatory Systems: The Main Difference between Insects and Mammals

**Figure 1:**
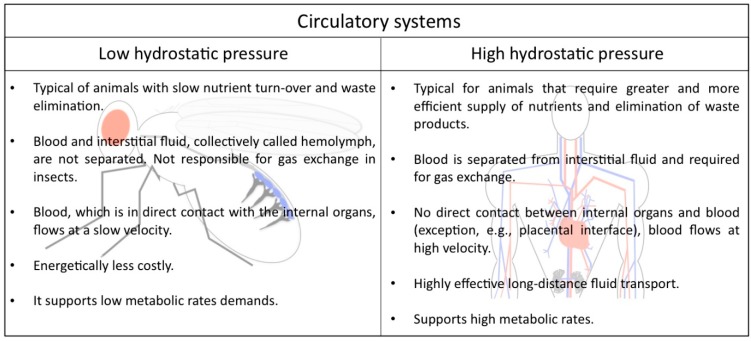
Comparison of the circulatory system in *Drosophila* and humans.

In the animal kingdom there are two main types of circulatory systems that are in concordance with the metabolic requirements of their host, suggesting that each kind of circuit offers specific advantages (Figure 1). Humans and other vertebrates, on the one hand, have a closed circulatory system, meaning that the blood is limited to vessels, never leaving the network of arteries, veins and capillaries, and is distinct from the interstitial fluid. Due to the elevated pressure that the blood generates inside the complex ramified network, this type of circulation is also called a high hydrostatic pressure system. It is a very efficient circuit that allows animals with high metabolic rates to stay active.

Lower hydrostatic pressure systems, on the other hand, such as those found in many mollusks and all arthropods, are commonly associated with open circulatory systems that require less energy to be built and maintained. *Drosophila* *melanogaster* displays such an open circulatory system, with a simple tube-like heart that pumps the hemolymph from the posterior body region towards the anterior. The hemolymph represents the interstitial fluid and is often called the insect “blood”, although it lacks the presence of oxygen-transporting blood cells. It directly supplies the organs with nutrients, signal peptides, and all kind of metabolites, hormones and macrophages responsible for wound healing. In addition, the hemolymph contains hemocytes that secrete extracellular matrix (ECM) proteins [3,4] and also contribute to the insect′s immune system. Gas exchange and transport is not the purpose of the heart, since insects harbor a dedicated tubular network, the tracheal system, for this purpose.

## 3. Histology of the Fly Heart

**Figure 2:**
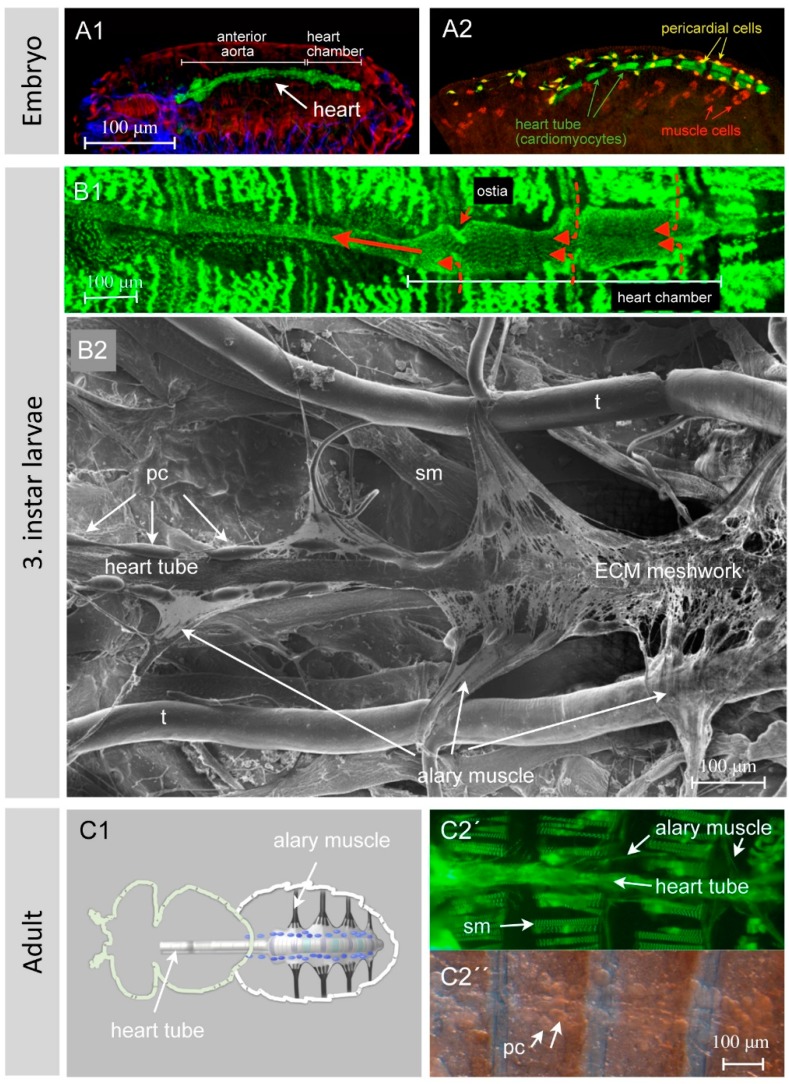
The *Drosophila* heart at different developmental stages: (**A1**) and (**A2**) show dorsoventral views of late wild-type embryonic hearts. Figure (**A1**) shows an immunostained embryo with three different organ systems labeled. Heart cells were visualized with an anti-GFP antibody to detect GFP expressed under the control of *hand*-GFP (green channel); somatic muscles were visualized with an antibody recognizing β3Tubulin (red channel); and the central nervous system with an anti-Mab22c10 antibody (blue channel). Further, (**A2**) shows a late embryo stained for GFP (green channel) and Even-skipped (red channel). The *Drosophila* line used for the immunostaining harbors a GFP expressed under the control of a truncated *hand* enhancer (*hand*-C^13-69^-GFP, Paululat personal communication), which drives GFP in all cardiomyocytes, except for those that form the ostia. Even-skipped is expressed in a subset of somatic muscles and pericardial cells. The pictures illustrate that the heart is composed of different cell types that can be distinguished by various molecular markers. Next, (**B1**) shows a semi-intact third instar larva expressing Zasp52 tagged with GFP [17]. The posterior heart chamber, with its wider diameter, and the ostia, through which the hemolymph enters the heart, are visible. Red arrows indicate the directionality of hemolymph flow. The reporter line also labels the somatic muscles. Also, (**B2**) shows a dissected wild-type third instar larva. Scanning electron microscope imaging was performed from the ventral side of the specimen; thus we look onto the ventral side of the larval heart, illustrating the heart with its alary muscles, pericardial cells and ECM network. Next, (**C1**) provides a schematic illustration of the location of the heart in an adult fly. The heart cells are color-coded to distinguish the cell types: grey: cardiomyocytes, violet: ostial cells, green: valve cells, blue: pericardial cells (nephrocytes), black: alary muscles. The ventral longitudinal muscles are not shown. Then, (**C2′**) shows an adult fly expressing Zasp66, tagged with GFP, in somatic muscles and cardiomyocytes [14]. For convenience, a corresponding brightfield picture is shown (**C2′′**). The methodologies used are: (**A1**,**A2**) Immunohistochemistry, (**B1**,**C2**) GFP fluorescence in a semi-intact animal, (**B2**) Scanning electron microscopy, (**C2′′**) Brightfield microscopy. Genotypes used: (**A1**,**B2**) wild type, (**A2**) *hand*-GFP transgene [6], (**C2**) Zasp66: GFP protein trap line [17]. Abbreviations: pc, pericardial cell; sm, somatic muscle; t, trachea. All pictures are oriented with the anterior side to the left.

**Figure 3:**
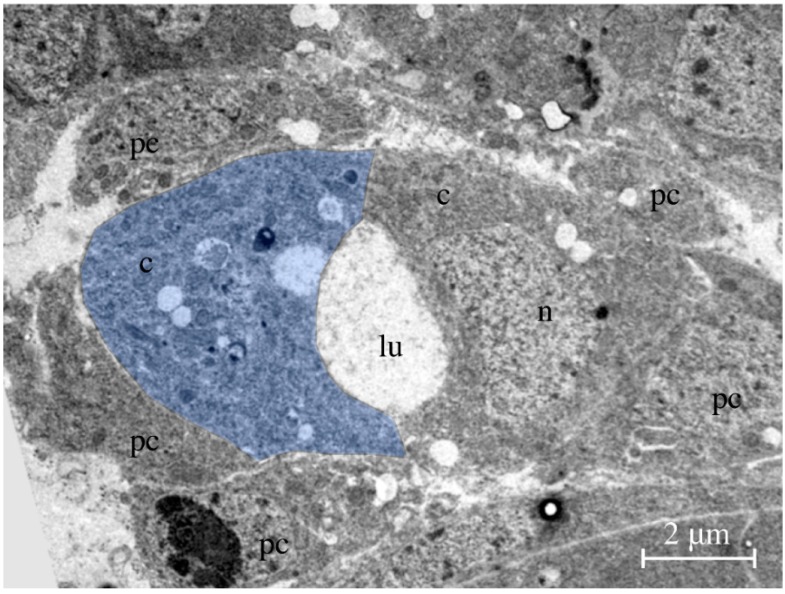
Transmission electron micrograph. The picture shows a cross-section through the heart of a late-stage 16 embryo. Two bean-shaped cardiomyocytes form the heart lumen. One of the two cardiomyocytes is labeled in blue. Abbreviations: c, cardiomyocyte; lu, lumen; n, nucleus; pc, pericardial cell.

The heart of *Drosophila*, also known as the dorsal vessel due to its spatial position underneath the dorsal epidermis (Figure 2(A1,A2)), is built early during embryogenesis by cardiomyocytes arranged in two opposing rows of cells that form a luminal space in between (Figure 3) [5]. At the end of embryogenesis, the heart begins beating. Contraction of cardiomyocytes pumps the hemolymph from the posterior heart chamber into the aorta, where it leaves the heart tube to spread into the open body cavity. During larval development, the animal grows dramatically and increases in length from 0.5 mm (embryo/first instar larva) to approximately 3–4 mm (third instar wandering larva). At the same time, the heart tube elongates exclusively through cell growth and not through cell proliferation, implying that the larval heart tube harbors the same number of cells as the embryonic heart (Figure 2(B1,B2)) [6]. Nevertheless, during larval growth a few new features arise: the luminal heart diameter expands dramatically, the cardiomyocytes grow in size as mentioned above, and many of the cells that are closely associated with the embryonic heart (pericardial cells, see below) disappear [7,8,9]. The adult heart (Figure 2(C1,C2)) differs in several anatomical and histological aspects from the larval heart due to specific differentiation processes taking place during metamorphosis [1,2,10]. These processes include, e.g., the formation of new incurrent openings (ostia) [11,12], the differentiation of additional intracardiac valves [6,8], the differentiation of the ventral longitudinal muscle (VLM) layer underneath the heart tube [13,14], the remodeling of the terminal heart chamber, and the formation of neuronal innervations [15,16].

## 4. Cell Types that Constitute the Fly Heart

The heart tube consists of different types of cardiomyocytes (see Section 4.1, Section 4.2 and Section 4.3) that account for the formation of one continuous tube-like heart lumen through which the insect′s “blood” streams. Furthermore, the *Drosophila* heart appears with associated pericardial cells that represent a crucial component of the fly′s excretory system (4.4). The heart tube is fixed in the open body cavity of the fly by alary muscles that connect the heart to the epidermis in a flexible manner (4.5). Pericardial cells, which belong to the excretory system of insects, accompany the heart tube (4.6). In adult flies, the ventral side of the heart is lined with a longitudinally orientated layer of syncytial muscles (4.4–4.6). Finally, an essential constituent of the cardiac system is composed of non-cellular tissue: the cardiac extracellular matrix (ECM) (4.7) that supports the heart′s architecture and functionality. We will briefly introduce the abovementioned cell types in the following sections.

### 4.1. Cardiomyocytes

Cardiomyocytes are cells with easily distinguishable functionalities form the heart tube; these are the contractile cardiomyocytes discussed in this Section (4.1), the ostia (4.2) and the intracardiac valve cells (4.3). Differentiation into different cells types is achieved early during embryogenesis by the activity of the so-called identity genes, e.g., T-box genes [18], Ladybird [19] or Tinman [20]. Identity genes have been studied intensively in the past and are still a major subject of ongoing research [21,22,23,24,25,26,27]. The above-mentioned work and other studies have led to a comprehensive understanding of the role of many transcriptional networks acting early during Drosophila cardiogenesis to ensure the proper diversification of cells originating from the cardiac primordia. In addition to the identity genes, which account for the later differentiation of single cardiomyocytes, it has been shown that the spatial organization of the heart tube along its anterior-posterior axis is regulated by the activity of homeotic genes, which define, e.g., the posterior heart chamber [24,28,29,30].

From anterior to posterior, the heart is divided into two regions. The anterior aorta displays a narrow luminal diameter and lacks incurrent openings during the embryonic and larval stages. The posterior heart chamber, which is separated from the aorta region by a pair of intracardiac valve cells, possesses a wider luminal diameter [8]. All cardiomyocytes that harbor sarcomeres are able to contract, as demonstrated by life cell video imaging. A couple of recent papers have shed light on the capability of cardiomyocytes to form a lumen in between the two opposing cells (reviewed in [31]), although many details remain unknown. Repellent-attractant proteins such as Slit [32,33,34,35,36], Robo [34,35,36], Unc [37], and Netrin [37], as well as in the Integrin-dependent coupling of ECM components [38,39] to the cardiomyocytes, are crucial for establishing and maintaining the luminal diameter. Nevertheless, all of these contractile cardiomyocytes are collectively responsible for the peristaltic movement of the heart wall that provides the propelling force for hemolymph flow within the cardiac lumen.

In *Drosophila* embryos and larvae, the heart tube is formed by 104 cardiomyocytes, and, due to histolysis and remodeling processes occurring during metamorphosis, this number is reduced to 84 in the adult fly [6]. All cardiomyocytes express characteristic muscle-specific sarcomere proteins such as Myosin, Zasp52 (Figure 2), or others, along with the transcription factors that potentially activate the expression of such proteins. Transcriptome [40,41,42,43] and proteomic [44] analyses based on dissected heart tissue or isolated cardiac cells have been successfully used to identify the repertoire of genes expressed in the *Drosophila* heart. These analyses led to the first insights into how cardiac genes are regulated on a global level.

### 4.2. Ostial Cells

The hemolymph of *Drosophila* enters the heart through the ostia, which are inflow openings formed by specialized cardiomyocytes. The embryonic and larval heart harbors three pairs of functional ostia located in the posterior heart chamber (Figure 2(B1)). They are thought to act passively like a bipartite clack valve. Adult flies, due to the structural changes to the heart chamber that occur during metamorphosis, display five pairs of ostia, which act in an identical manner [8,11,12,18,29,45,46]. The ostial cells are colored lilac in Figure 2(C1). It has been reported for several insects that heartbeat reversal eventually leads to retrograde (from anterior to posterior) hemolymph flow [47,48,49]. It is not clear yet whether a terminal opening in the *Drosophila* heart allows such a retrograde outflow, but evidence for this has been uncovered by anatomical studies [12] and immunostainings of ostia-specific markers [10]. Whether these terminal cells of the adult fly heart indeed act as flaps or exert other, yet unknown functions remains to be elucidated.

### 4.3. Intracardiac Valves

**Figure 4:**
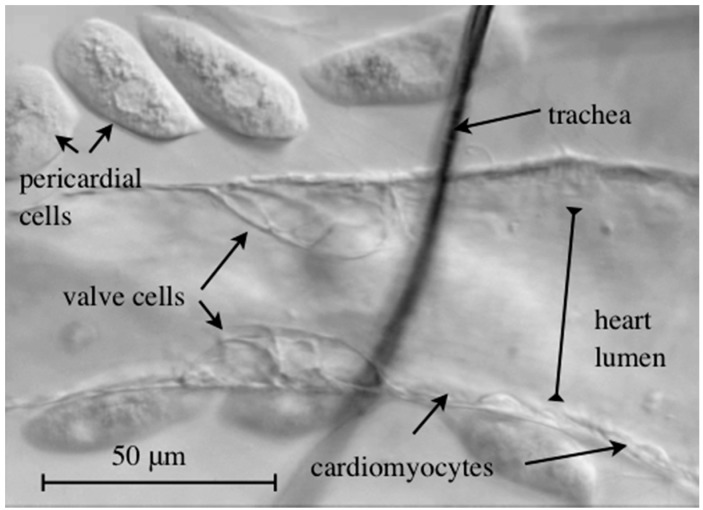
Brightfield microscopy image of a semi-intact wild-type third instar larval heart. The *Drosophila* heart harbors two specialized valve cells that regulate blood flow directionality.

Directionality of blood and lymph flow in vertebrates is regulated by valves present in the heart and the veins, and within the vessels of the lymphatic system. In mammals, valves that separate the heart chambers consist of a core of connective tissue with collagen and elastin as the major constituents, whereas venous valves are cellular flaps that are lined with a rather thin matrix [50]. Valves regulate unidirectional flow when the leaflets of the valve flip to the center of the vessel and thereby close the luminal space. The *Drosophila* heart has one (in larvae) or three (in adults) intracardiac valves that subdivide the heart into distinct chambers. In Figure 2(C1), the valves are colored in green. Each valve consists of two contralaterally located cells with a unique histology (Figure 4). Large intracellular vesicles occupy most of the cell′s volume and may cause the roundish shape of the cell, thereby enabling them to close the cardiac luminal space and block hemolymph back-flow. Interestingly, neither differentiation nor the biomechanical functionality of valve cells has been described in detail so far, as indicated by the limited number of publications that look directly at these cells [8,43,51].

### 4.4. Alary Muscles

Upon crawling, peristaltic contraction waves run along the highly flexible larval body, necessitating a flexible heart suspension system. This is provided by the alary muscles (Figure 2(B2,C1,C2)), which span from epidermal attachments towards the heart and maintain the heart tube in an anatomically correct position [2,52,53]. In larvae, seven pairs of alary muscles contact the heart tube indirectly, mediated by an interface formed by extracellular matrix components. Adult flies possess only four pairs of alary muscles, the posterior ones that escape from metamorphosis (colored black in Figure 2(C1)). Alary muscles arise from single myoblasts specified by the combined activity of identity genes [13,54,55]. These eventually fuse and form syncytial alary muscles containing five to six nuclei.

### 4.5. Pericardial Cells

The pericardial cells in the late larva (Figure 2(B2)) and adult fly (Figure 2C′′), together with the garland cells and the malpighian tubules, form the excretory system of the fly. Mononucleated pericardial cells, as well as the binucleated garland cells, are highly endocytic and are responsible for the removal of effete cells, dispensable material and macromolecules [56,57]. Recent studies have demonstrated that the insect′s pericardial cells represent nephrocytes, analogous to reticuloendothelial cells in mammals, and thus act as hemolymph/blood filtration systems [56,57,58,59,60].

**Figure 5:**
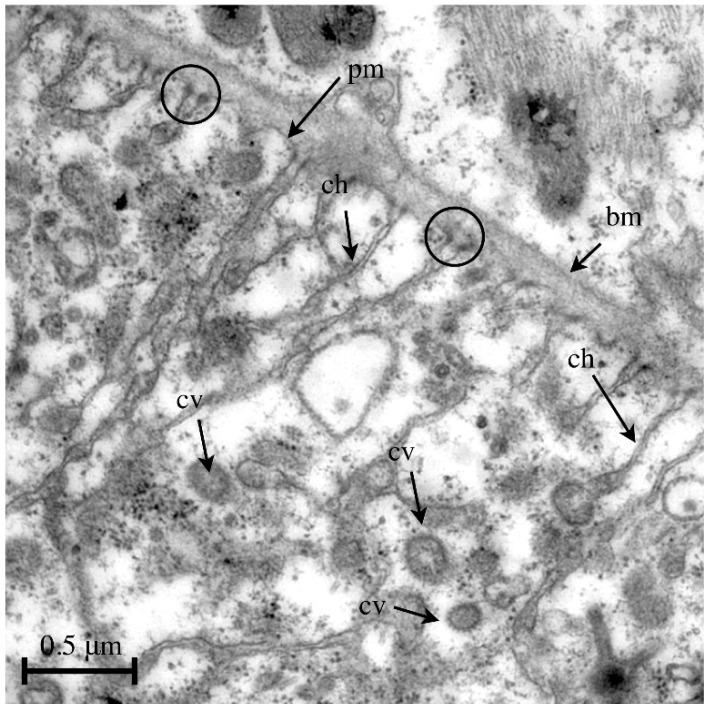
Transmission electron micrograph of the periphery of a pericardial cell isolated from a wild-type third instar larva. The cortex of pericardial cells is characterized by invaginations of the plasma membrane, forming a labyrinth-like channel system with a considerably increased surface area. Endocytosis takes places at the terminal sites of the channel system. Such an event is visible at the labeled channel on the right. Abbreviations: bm, basement membrane; pm, plasma membrane; ch, labyrinth channel; cv, coated vesicle; slit diaphragms are indicated by circles.

In *Drosophila*, the pericardial cells are located close to the heart tube, often near the incurrent openings that are ideally placed to filtrate the bypassing hemolymph. Ultrastructural analyses have shown that post-embryonic pericardial cells display characteristic slit diaphragms at the entry points to the labyrinth-like channel system (surface expansion) (Figure 5) [8,56,57,61]. A second role for pericardial cells has been identified recently: they act as sensors for oxidative stress [62]. Interestingly, the number of pericardial cells in the embryo is much higher than in larvae or adults, and all of these cells essentially lack the diaphragm system, indicating that the embryonic pericardial cells represent not yet fully differentiated nephrocytes and thereby might fulfill other functions. This has been shown, e.g., in a small subpopulation of eight pericardial cells that develop into wing hearts [63]. Additionally, the embryonic pericardial cells express and secrete ECM proteins, thereby contributing, together with other tissues such as adipocytes and hemocytes, to the production of basement membrane material [64]. Finally, the presence of certain peptidases at the surface of Even-skipped-positive pericardial cells suggests that this cell type is also involved in regulating homeostasis of hemolymph-circulating peptides [65].

### 4.6. Ventral Longitudinal Muscles

**Figure 6:**
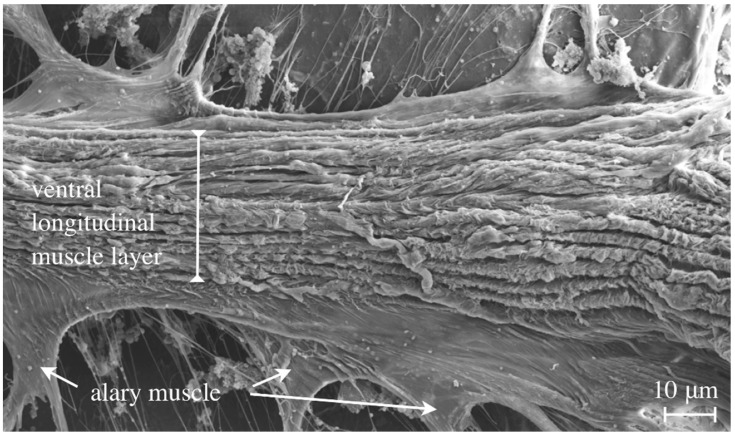
An adult fly was dissected, prepared for Scanning-EM and viewed from the ventral side (inside-out) to demonstrate the layer of ventral longitudinal muscles that run from anterior to posterior underneath the heart tube.

The ventral longitudinal muscles represent a special layer of syncytial somatic muscles that are located beneath the heart and exist only in adult flies (Figure 6) [1,8,13,14]. It has been suggested that this muscle layer contributes to the dorsal diaphragm, an insect tissue consisting of connective tissue (ECM) and somatic muscles that separates the pericardial sinus (where the heart tube is located) from the abdominal body cavity. Such a sinus might account for an optimized diastolic and systolic hemolymph flow, although this has not been investigated in detail so far. The developmental origin of the ventral longitudinal muscles remained unknown for a long time, although it was shown earlier that this type of muscle is syncytial, indicating similarities to the somatic body wall muscles and alary muscles in flies. Schaub and colleagues showed recently that the ventral longitudinal muscles originate from a quite unexpected developmental mechanism. The first three pairs of the larval alary muscles dedifferentiate into mononucleated myoblasts. These cells act as muscle founders that recruit cells for fusion from a pool of fusion-competent myoblasts. It is assumed that differentiation of the ventral longitudinal muscles is orchestrated by instructive cues from neighboring tissue, presumably from the heart [13].

### 4.7. Connective Tissue—ECM

**Figure 7:**
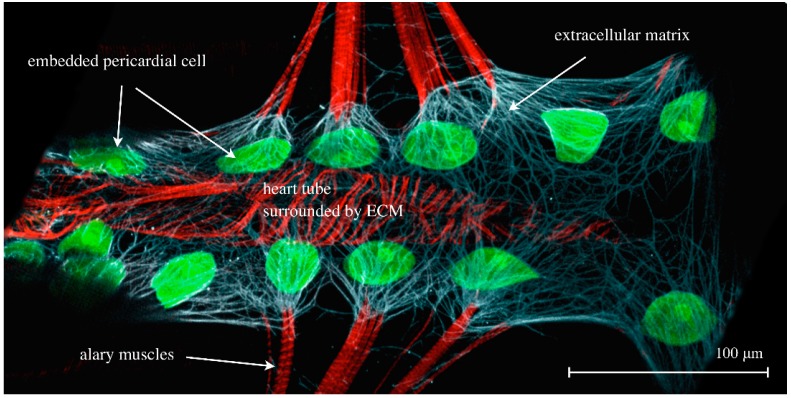
The cardiac ECM of a dissected third instar larva expressing *hand*-GFP (green channel) and stained for Phalloidin (F-actin, muscles, red channel) and anti-Pericardin, a cardiac-specific ECM constituent (white channel).

The cardiac extracellular matrix is a non-cellular fundamental constituent of the circulatory system (Figure 7). Recent work on the collagen IV-like structural protein Pericardin and its cardiac recruitment factor Lonely heart (ADAMTSL6) has demonstrated that cardiac matrices that fail to incorporate and assemble Pericardin become unstable upon aging, which finally results in heart collapse and heart failure [64]. Similar results were obtained in mutants that affect LamininB2 [66], or LamininB1 and Cg25c (Collagen IVa1) [67], components that are collectively required to establish the cardiac matrix. As illustrated in Figure 2(B2) and Figure 7, the cardiac matrix in *Drosophila* forms a cage-like network that covers the heart tube entirely. Nephrocytes are embedded in this meshwork, which is formed from the fibrous network of collagen, Pericardin and other proteins [64]. It is assumed that one of the important functions of the cardiac ECM is to position the nephrocytes close to the bypassing hemolymph so as to ensure easy accessibility. This might enhance the filtration function of nephrocytes because the hemolymph must pass through this area before entering the heart lumen. Moreover, the cardiac ECM connects the counter-lateral alary muscles with the heart tube and contributes to the dorsal diaphragm, which eventually subdivides the body cavity into separate pressure areas, thus supporting diastolic refilling of the heart. We propose that the highly mobile architecture of the heart tube is essentially achieved via a flexible suspension of the heart that is in turn facilitated by the ECM-mediated alary muscle connection. Furthermore, the composition of the cardiac ECM directly influences the biomechanical properties of the heart. Similar to the mammalian vascular system, in which components such as Elastin regulate the elasticity of the vessel wall, distinct structural components of the *Drosophila* cardiac ECM might play similar roles [64,68,69,70,71,72].

## 5. Accessory Hearts

Hemolymph circulation in *Drosophila* is driven by the orchestrated activity of several organs and tissues. The main organ is the dorsal vessel that pumps the hemolymph from posterior to anterior. The hemolymph is further circulated by the activity of the body wall muscles situated in the open body cavity. Interestingly, the insect′s circulatory system also comprises additional pulsatile organs (“hearts”) that support hemolymph exchange in the insect′s appendages, such as the legs, the wings and the antenna [73,74]. It has been shown that these additional pulsatile organs are required not only for maintaining the proper physiological conditions within the long appendages (e.g., legs, wings) by ensuring constant renewal of the hemolymph, but also for the development and functionality of organs (wings) [63,75,76,77]. Although only a side aspect herein, the additional pulsatile organs supporting the wings of the fly (*i.e.*, the wing hearts) are differentiated from a subset of eight embryonic pericardial cells [63].

## 6. Summary

In recent years, numerous papers have been published emphasizing the adult heart of *Drosophila* as an excellent human disease model by which to study, e.g., the principles of arrhythmia [78], cardiac aging [79] or obesity [80,81], to name but a few. In the *Drosophila* heart model, it is possible to manipulate cell specification, cell differentiation, organ architecture, heart physiology, *etc.*, in order to study the consequences of such manipulations in a living animal. It has been demonstrated that even severe malformation or malfunction of the cardiac system does not necessarily lead to the animal′s death, at least under laboratory animal husbandry conditions [64,82,83]. This allow researchers to study aspects of heart differentiation, heart organogenesis and heart performance, which are not accessible at all in other animal models due to the fact that genetic or pharmaceutical manipulations often cause early lethality, e.g., in mammals. In this paper, we have presented a short overview of the histology and anatomy of the *Drosophila* heart, with a focus on larval and adult tissue.
